# A novel method for multifactorial bio-chemical experiments design based on combinational design theory

**DOI:** 10.1371/journal.pone.0186853

**Published:** 2017-11-02

**Authors:** Xun Wang, Beibei Sun, Boyang Liu, Yaping Fu, Pan Zheng

**Affiliations:** 1 College of Computer and Communication Engineering, China University of Petroleum, Qingdao 266580, Shandong, China; 2 State-owned Asset and Laboratory Management Department, China University of Petroleum, Qingdao 266580, Shandong, China; 3 Institute of Complexity Science, Qingdao University, Qingdao 266071, Shandong, China; 4 Faculty of Engineering, Computing and Science, Swinburne University of Technology Sarawak Campus, Kuching 93350, Malaysia; Xiamen University, CHINA

## Abstract

Experimental design focuses on describing or explaining the multifactorial interactions that are hypothesized to reflect the variation. The design introduces conditions that may directly affect the variation, where particular conditions are purposely selected for observation. Combinatorial design theory deals with the existence, construction and properties of systems of finite sets whose arrangements satisfy generalized concepts of balance and/or symmetry. In this work, borrowing the concept of “balance” in combinatorial design theory, a novel method for multifactorial bio-chemical experiments design is proposed, where balanced templates in combinational design are used to select the conditions for observation. Balanced experimental data that covers all the influencing factors of experiments can be obtianed for further processing, such as training set for machine learning models. Finally, a software based on the proposed method is developed for designing experiments with covering influencing factors a certain number of times.

## Introduction

The design of experiments, also known as experimental designs, deal with the task of finding relationship among the influencing factors of multifactorial experiments as well as the contribution of each factors to the outcome [1–3]. Experimental design refers to how participants are allocated to the different conditions in an experiment. It involves not only the selection of suitable predictors and outcomes, but planning the delivery of the experiment under statistically optimal conditions given the constraints of available resources [4, 5]. The aim is to design a number of experiments for predicting the outcome by introducing a change of the preconditions. With the experimental data, mathematical models might be built to calculate the outcome from variables or experimental conditions. The three main concerns in experimental design are validity, reliability, and replicability [6, 7]. The mostly used experimental design methods include Plackett-Burman designs [8], frequentist and Bayesian based approaches [9], response surface methodology [10], central composite design [11] and so on.

In recent years, machine learning methods are used to modelling the bio-chemical experiments with a set of experimental data, see e.g. deep neural network [12], spiking neural P systems [13–17]. Recently, many significant artificial intelligent algorithms and data processing strategies has been applied on data mining, such as a self-adaptive artificial bee colony algorithm based on global best for global optimization [18], the public auditing protocol with novel dynamic structure for cloud data [19], privacy-preserving smart semantic search method for conceptual graphs over encrypted outsourced data [20], a privacy-preserving and copy-deterrence content for image data processing with retrieval scheme in cloud computing [21], and machine learning method have been applied for experimental condition design, see. e.g. a secure and dynamic multi-keyword ranked search scheme over encrypted cloud data [22].

The general idea is to learn from the experimental data, and then achieving a prediction model of the experiment, which matches the known data in a acceptable level. With the model, some optimal conditions for maximizing the outcome or minimizing the cost can be obtained [23–26]. However, the experimental data obtained or collected by classical experimental design methods, such as Plackett-Burman designs, response surface methodology, central composite design, the data is quite unbalanced, are not balanced well, such that we cannot get well fitting models by using machine learning methods.

Combinatorial design theory belongs the field of combinatorial mathematics, which deals with the existence [27–29], construction and properties of systems of finite sets with generalized concepts of balance and/or symmetry [30–32]. It is formulated in [33] that combinatorial designs can provide potential tools in the area of design of experiments, particularly for the design of biological experiments.

In this work, borrowing the concept of “balance” in combinatorial design theory, we propose a novel method for multifactorial bio-chemical experiments design. In the method, balanced templates from the existence and construction in combinational design theory are used to select the experiments which should be done for conditions observation. We can get balanced experimental data from the experiments selected that covers all the influencing factors of experiments for further processing, particularly for machine learning based modelling. Finally, a software with the proposed method is developed, which provides a simulation tool for designing multifactorial experiments, by which the designed experiments can cover influencing factors a pre-designed number of times.

## Methods

In this section, we introduce the method proposed for experimental design based on combinational design theory.

Before introducing the method, we clarify the meaning of some involved symbols. Let *m* be the number of influencing factors for an experiment. These factors are denoted by *f*_1_, *f*_2_, …, *f*_*m*_. By *n*_*j*_ with *j* = 1, 2, …, *m*, we denote the possible values of factor *f*_*j*_. Parameter *s* is set to be covering times of all the factors. Among the *n*_*j*_ values of any factor *f*_*j*_, it is not necessary to select all the values, but *t* values is sufficient to cover all the cases of factors. The number of experiments needed to cover all the cases of factors and all the values is denoted by *r*. In other word, we need design *r* experiments to cover all the factors *s* times with each time considering *t* values of any factor.

### The mathematical model of experimental design

Let *P* be a *m* × *n* matrix recording the *m* influencing factors, where *n* = max{*n*_1_, *n*_2_, …, *n*_*m*_}. Mathematically, matrix *P*_*m*×*n*_ = (*p*_*ij*_)_*m*×*n*_ is denoted as follows

$$\begin{array}{c} P=[\begin{array}{ccccc} p_{11} & p_{12} & p_{13} & \cdots & p_{1n}\\ p_{21} & p_{22} & p_{23} & \cdots & p_{2n}\\ \vdots & \vdots & \vdots & \ddots & \vdots \\ p_{m1} & p_{m2} & p_{m3} & \cdots & p_{mn} \end{array}]. \end{array}$$

By *p*_*ij*_, we denote the the *j*th possible value of the *i*th influencing factor to the experiment. The problem of experimental design is to find suitable values of *r*,*s* and *t*, with which we can obtain an experiment design, i.e., a group of experiments, *γ* = {*γ*_1_, *γ*_2_, …, *γ*_*g*_}. Each experiment *γ*_*k*_ ∈ *γ* is of the form *γ*_*k*_ = {*p*_1*k*_1__, *p*_2*k*_2__, …, *p*_*mk*_*m*__}. The experimental design to be found should satisfy the following items:

- ${⋃}_{k=1}^{g}\gamma_{k}=\left\{p_{ij}|i=1,2,\ldots ,m,j\;=1,2,\ldots ,n\right\}$;
- for any *γ*_*k*_ = {*p*_1*k*_1__, *p*_2*k*_2__, …, *p*_*mk*_*m*__}, its elements are from matrix *P*, where *p*_*jk*_*j*__ is elected from the *j*th row of matrix *P* with *j* = 1, 2, …, *m*.
- in total *g* × *m*, that is, *r* experiments in the design;
- given *t* elemental elements (crucial to the experiment) from matrix *P*, and these elements present in *γ* for *s* times.

When given the *t* elemental elements, the object is to finding certain experiment design *γ*, which has minimal value of *r* and maximal value of *s*.

### Theoretical support from combinational design theory

With the notations defined above, the experimental design can be transferred as finding a suitable values and combination of parameters *t*, *s* and λ. In combinational design theory, the concept of difference set have some common features with parameters *t*, *s* and λ. This provides a way to design experiments by the way of finding difference set developed in combinational design theory. Let’s briefly recall some basic concepts of difference set.

Let *G* be an Abelian group in modern algebra theory. A (*v*, *k*, λ) difference set is a subset *D* of a group *G* such that the order of *G* is *v*, the size of *D* is *k*, and every nonidentity element of *G* can be expressed as a product $d_{1}d_{2}^{-1}$ of elements of *D* in exactly λ ways (when *G* can be written with a multiplicative operation) [34]. For any element *g* in *G*, if subset *D* is a difference set, then it holds *g* ⋅ *D* = {*g* ⋅ *d*: *d* ∈ *D*} is also a difference set, which is named as a translate of *D*. It is known that the set of all translates of a difference set *D* can achieve a symmetric block design. In such a design there are *v* elements and *v* blocks. In each block of the design, it has *k* points, and each point is contained in *k* blocks [30].

In combinational design theory, there are some theorems on designing difference sets with distinguished values of parameters *v*, *k* and λ). With the concepts of difference set in combinational design theory, it is not hard to find that finding suitable and reasonable values of *s*, *t* and *r* is similar of designing a different set. The strategy used in constructing different sets can be used to determine values of *r*, *s*, and *t*, thus achieving a way for experimental design.

### The algorithm for experimental design

**Fig 1:**
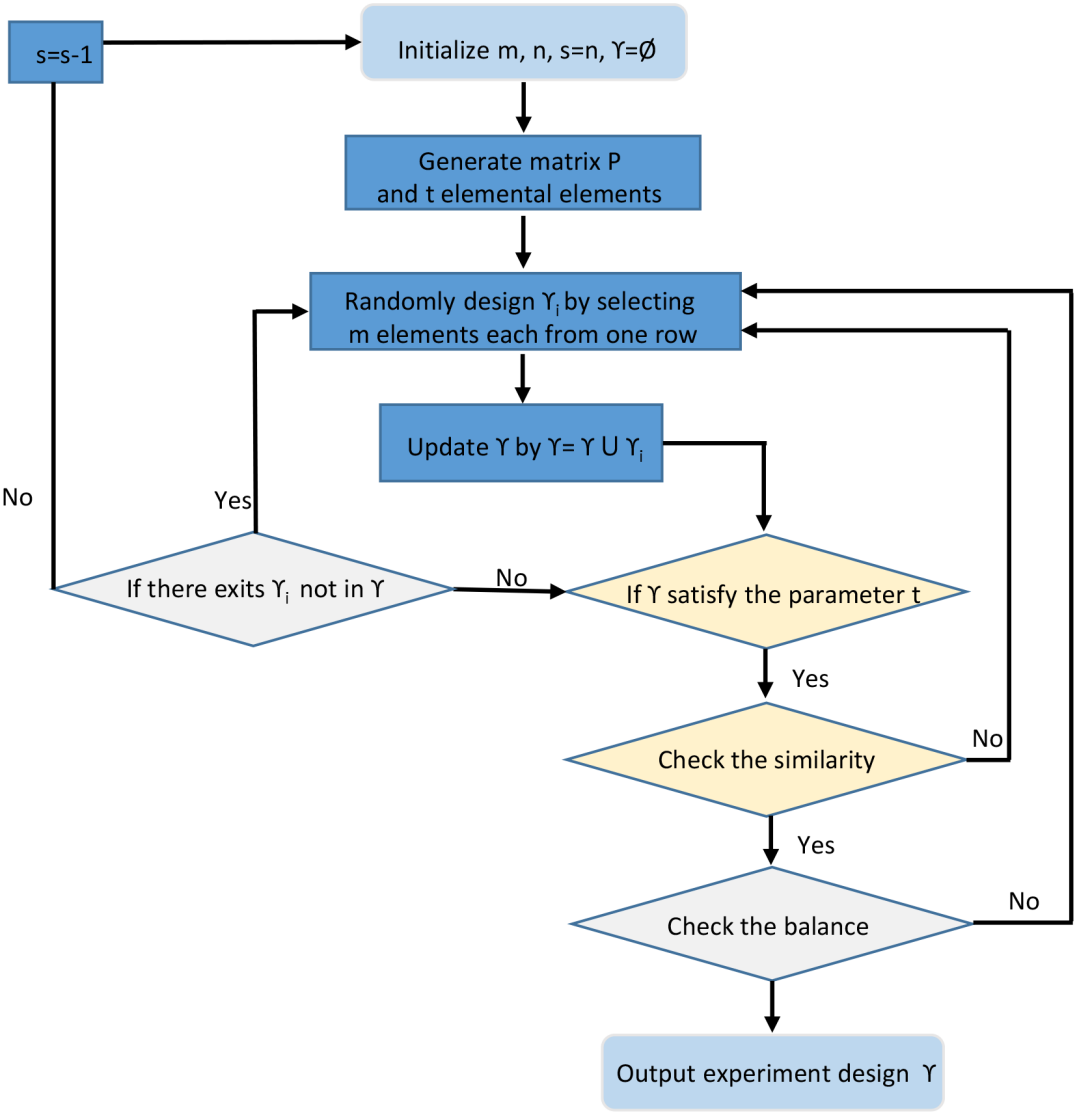
The flowchart of the algorithm.

In this subsection, we propose an algorithm for finding suitable values of parameters *r*, *s*, and *t*, like finding different sets in combinational design theory. The flowchart of the algorithm is shown in Fig 1.

The general process of the algorithm is as follows.

**Step 1**. Initialize parameters *m*, *n* and set *s* = *n*, *γ* = ∅;
**Step 2**. Generating matrix *P*_*m*×*n*_ with the *n* values of each of the *m* influencing factors.
**Step 3**. Selecting the *t* elemental elements from matrix *P*.
**Step 4**. Generating *γ*_*i*_ by randomly select one element from each row of matrix *P*.
**Step 5**. Updating *γ* = *γ* ∪ *γ*_*i*_.
**Step 6**. If there exits some other *γ*_*i*_ that can be generated, then go to **Step 4**.; otherwise go to **Step 7**.
**Step 7**. Check if the generated *γ* covers all the *t* pro-defined elemental elements of matrix *P*. If so, go to **Step 8**.; otherwise, updating *s* = *n* − 1 and go to **Step 1**. to repeat the process.
**Step 8**. Check if the generated *γ* matches the request of similarly by comparing any two groups of experiments. If so, go to **Step 9**.; otherwise, go to **Step 4**. to re-design the experiments.
**Step 9**. Check if the generated *γ* matches the request of balance, which is calculated by the rate between the minimal times and maximal times of the pairs of two influencing factors in the designed experiments. If so, halt the algorithm and output the designed experiments *γ*; otherwise, go to **Step 4**. to re-design the experiments.

## Simulation tools

**Fig 2:**
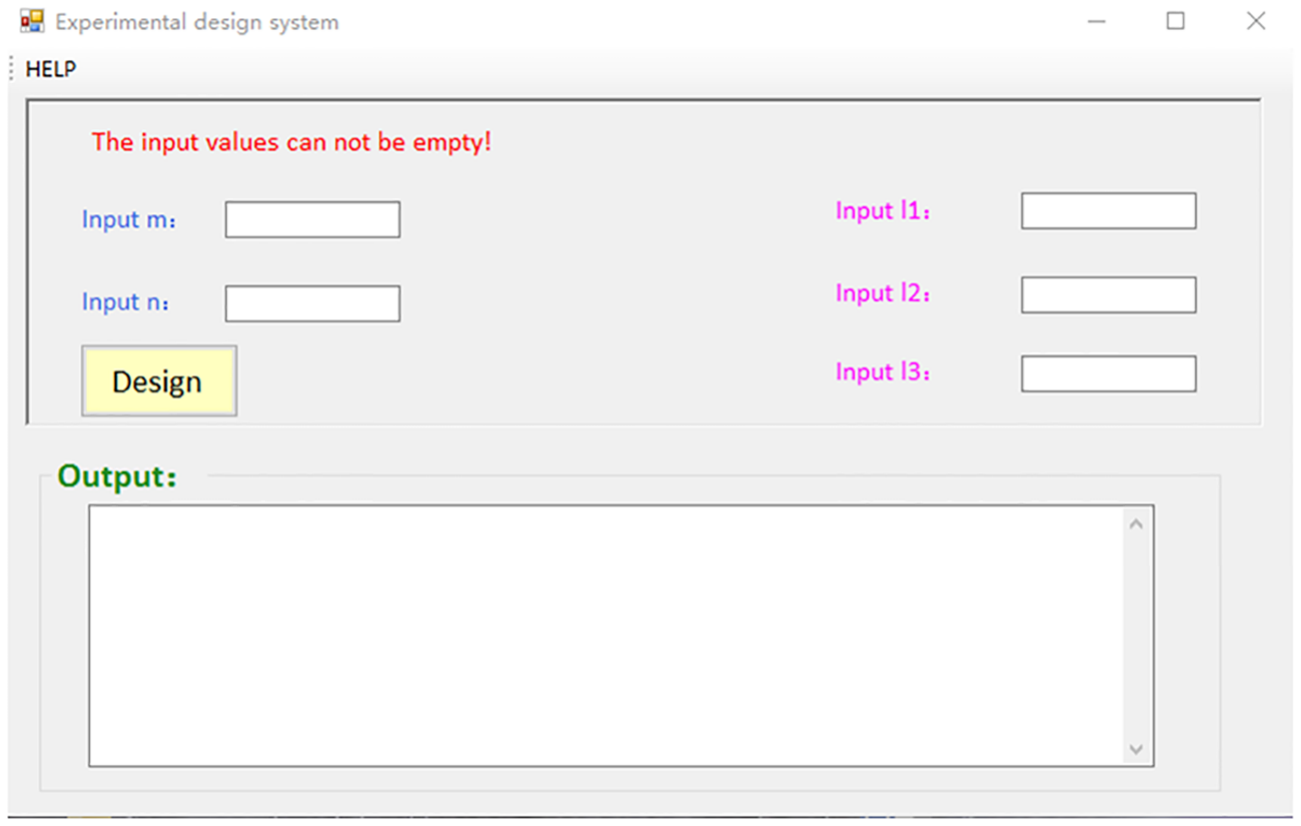
The starting page of the simulation software.

A software based on Visual Studio 2010 is developed for the the simulation of the proposed algorithm. The simulation tool produces a group of experiments with generalized concepts of balance and/or symmetry in combinational design theory. We set a similarly comparison mechanism to avoiding similar groups of experiments are designed. The starting page of the software is shown in Fig 2.

The meaning of the parameters in Fig 2 is as follows.

- The number of influencing factors is represented by *m*.
- The maximal number of possible values of all the influencing factor is *n*.
- Input 11 is the value of the minimal similarity between any couple of *γ*_*i*_ and *γ*_*j*_ in the designed *γ*. If there exists any two groups of designed experiments having similarity less the value of Input 11, then it repeats the algorithm to generate a new group of experiments.
- Input 12 is the number of total experiments that need be designed.
- Input 13 is the balance measure of the designed experiments, which is calculated by the rate between the minimal times and maximal times of the pairs of two influencing factors in the designed experiments.

**Fig 3:**
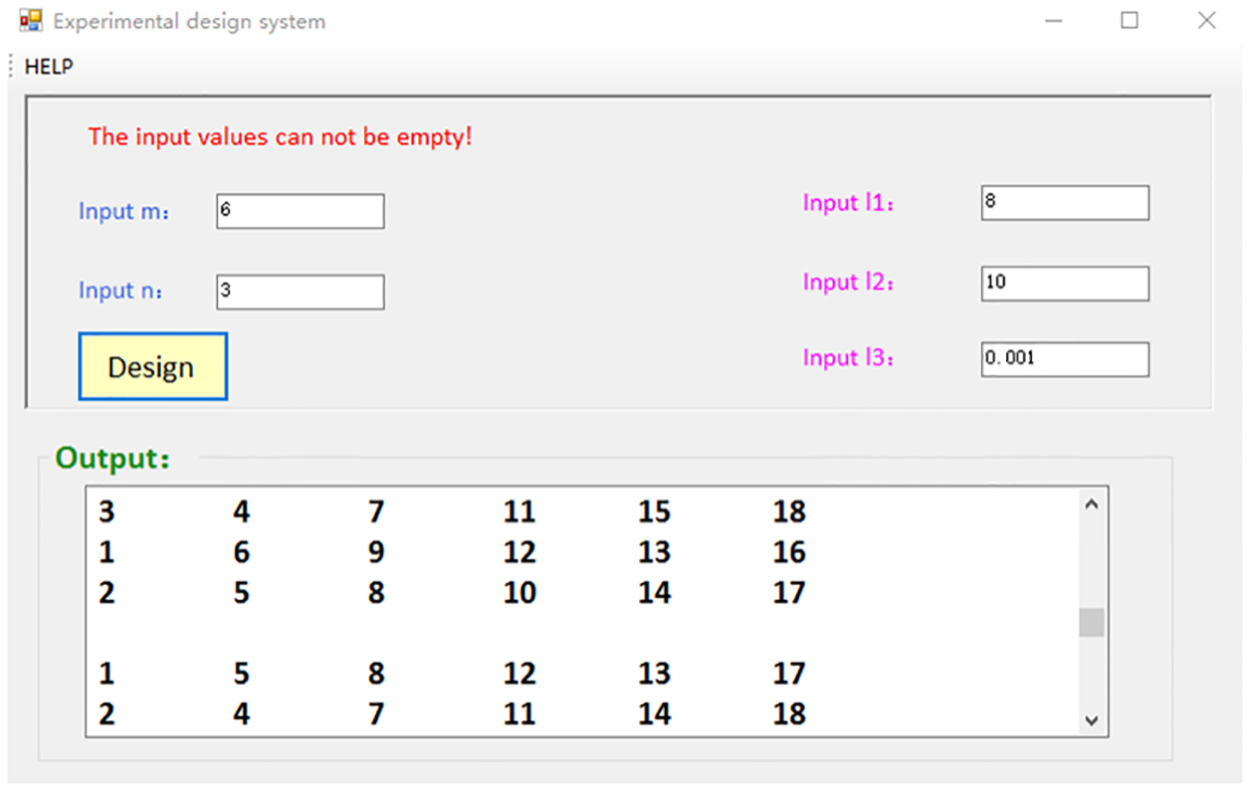
An example with inputs *m* = 6, *n* = 3, Input 11 be 8, Input 12 be 10 and Input 13 be 0.001.

For example, we can design experiments with *m* = 6, *n* = 3, Input 11 be 8, Input 12 be 10 and Input 13 be 0.001. The salutation page is shown in Fig 3.

The simulation result can be used to design experiment of fungal fermentation experiments. There are 6 influencing factors: (1) inoculum concentration, (2) the volume of liquid, (3) temperature, (4) PH value, (5) yeast concentration and (6) amylaceum concentration. Each influencing factor has 3 possible values, which is shown in Table 1.

**Table 1:** Possible values of the influencing factors.

inoculum concentration	the volume of liquid	temperature	PH value	yeast	amylaceum
5g/L	50ml	27.5°C	6	4g/L	45g/L
6g/L	75ml	30°C	7	6g/L	70g/L
7g/L	100ml	32.5°C	8	8g/L	85g/L

Using the simulation result of *γ*, we can obtain the designed experiments as follows.

- *γ*_1_ = {{5*g*/*L*, 75*ml*, 32.5°*C*, 7, 4*g*/*L*, 70*g*/*L*}, {6*g*/*L*, 50*ml*, 30°*C*, 6, 8*g*/*L*, 45*g*/*L*}, {7*g*/*L*, 100*ml*, 27.5°*C*, 8, 6*g*/*L*, 85*g*/*L*}}.
- *γ*_2_ = {{5*g*/*L*, 100*ml*, 30°*C*, 8, 6*g*/*L*, 45*g*/*L*}, {6*g*/*L*, 75*ml*, 27.5°*C*, 7, 4*g*/*L*, 85*g*/*L*}, {7*g*/*L*, 50*ml*, 32.5°*C*, 6, 8*g*/*L*, 70*g*/*L*}}.
- *γ*_3_ = {{5*g*/*L*, 50*ml*, 27.5°*C*, 6, 8*g*/*L*, 85*g*/*L*}, {6*g*/*L*, 100*ml*, 32.5°*C*, 8, 6*g*/*L*, 70*g*/*L*}, {7*g*/*L*, 75*ml*, 30°*C*, 7, 4*g*/*L*, 45*g*/*L*}}.

The similarity of each pair of *γ*_*i*_ and *γ*_*j*_ with *i* ≠ *j* and *i*, *j* ∈ {1, 2, 3} is less than 0.001. The balance measure of the designed experiments is 1/3.

## Conclusion

In this work, we propose a novel method for multifactorial experiments design, where the generated concept of “balance” in combinatorial design theory is considered. In our method, balance and similarity are both used to select the experiments which should be done for conditions observation, with which we can get balanced experimental data from the experiments selected that covers all the influencing factors of experiments for further processing.

Since there is theoretical support in combinational design theory to ensure the exists of certain combination of parameters, our algorithm just starts from a random point and proceeds to find the possible designed experiments. By checking the similarity and balance designed experiments, we can choose to repeat the algorithm or output the desired experimental design. In complexity theory, starting from a random point would bring some extra computation consumption. It is of interest to design intelligent algorithms, such as GA with fitness function relating the similarity and balance, to improve the performance of our method.

For further research, some newly developed evolution computing models and algorithms, see e.g. [35–43], can be used to improve the balanced data sampling method. As well learning and training requests on the data for neural-like computing models and spiking neural networks [14, 44] should be considered in design balanced templates. In DNA computing, it needs to design DNA probes and DNA sequences [45–48], in which balanced template might provide useful tools. As well, some recently developed data processing and mining methods, such as the speculative approach to spatial-temporal efficiency for multi-objective optimization in cloud data and computing [49], privacy-preserving smart similarity search methods in simhash over encrypted data in cloud computing [49], k-degree anonymity with vertex and edge modification algorithm [50], kernel quaternion principal component analysis for object recognition [51], might be used for optimizing experiment design with intelligent methods.
